# Post-ischemic modification of neurogenesis and oligodendrogenesis in rodent models

**DOI:** 10.3389/fncir.2026.1803118

**Published:** 2026-04-16

**Authors:** Yoshihide Sehara, Shinya Mochizuki, Reiji Yamazaki

**Affiliations:** 1 Division of Genetic Therapeutics, Center for Molecular Medicine, Jichi Medical University, Shimotsuke, Japan; 2 Department of Cell Biology and Anatomy, Jichi Medical University, Shimotsuke, Japan; 3 Department of Anatomy, Division of Histology and Cell Biology, School of Medicine, Jichi Medical University, Shimotsuke, Japan

**Keywords:** ischemia, neural stem/progenitor cells, neurogenesis, oligodendrogenesis, stroke

## Abstract

Neurogenesis and oligodendrogenesis occur throughout life under both physiological and pathophysiological conditions. Brain insults such as ischemia, trauma, epilepsy, or Alzheimer disease result in the promotion of neurogenesis and oligodendrogenesis; however, the mechanisms and the roles of this promotion are not well elucidated. Neurogenesis occurs in two distinct regions in the brain, namely, the subventricular zone (SVZ) of the lateral ventricle and the subgranular zone (SGZ) of the dentate gyrus. Neural stem cells (NSCs) have the potential to self-renew, proliferate, and differentiate into various cell types. NSCs in the SVZ migrate toward the site of injury, and those in the SGZ migrate toward the granule cell layer after ischemic insult. Numerous animal experiments have shown that inhibition of post-ischemic neurogenesis both in the SVZ and the dentate gyrus impairs functional recovery. Oligodendrogenesis regenerates myelin around demyelinated axons after white matter injury, thus promoting functional recovery after ischemia. Oligodendrocyte progenitor cells derived from NSCs and progenitor cells of the SVZ and from intrinsic cells from other brain regions proliferate at the demyelinated lesions. However, deposition of extracellular matrices, including chondroitin sulfate proteoglycans, hyaluronan, fibronectin, and fibrinogen, have been reported to inhibit remyelination. Furthermore, our data showed that type I collagen was deposited in the white matter lesions of stroke patients, and that it may inhibit oligodendrocyte differentiation in these lesions. In this review, we focus on the mechanisms and the roles of post-ischemic neurogenesis and oligodendrogenesis based on recently published data of mainly rodent models.

## 1 Introduction

The brain is a vulnerable organ that can be irreversibly damaged by relatively mild insults, such as ischemia, trauma, and epilepsy (Fisher and Savitz, 2022; Tang et al., 2023). Cerebral ischemia is one of the major causes of death and disability worldwide, and it is associated with motor and cognitive deficits (Schneider et al., 2023). Cerebral ischemia can be broadly categorized into two groups: (1) global ischemia in which cardiac arrest transiently causes complete depletion of blood supply to the entire brain; (2) focal ischemia in which a thrombus occludes a proximal region of the cerebral or carotid artery, which causes depletion of blood supply to the distal area. Subsequent to ischemic insult, complex pathophysiological cascades such as inflammatory response, oxidative stress, mitochondrial dysfunction, blood–brain barrier damage, vascular regeneration, and programmed cell death are induced (Meng et al., 2025). Following ischemic damage, endogenous neurogenesis and oligodendrogenesis are promoted, both of which have been shown to contribute to functional recovery (Modi et al., 2020; Yamazaki et al., 2021; Youssef et al., 2021; Gao et al., 2023; Jin et al., 2023).

Rodent models are indispensable for revealing the pathophysiology of post-ischemic neurogenesis and oligodendrogenesis. For global ischemia, the 4-vessel occlusion model in rats and mice, in which bilateral carotid and vertebral arteries are transiently occluded, reproduces the pathophysiology of cardiac arrest caused by arrythmia, choking, and drowning. In the gerbil model of forebrain ischemia, transient occlusion of bilateral carotid arteries is sufficient to reproduce the post-ischemic neuronal death and neurogenesis in the hippocampus, because the collateral blood supply from the posterior circulation is insufficient (Yoo et al., 2016; Kim et al., 2020; Sehara et al., 2024). One of the most common models of focal ischemia is the transient middle cerebral artery occlusion (tMCAO) mouse model, in which a microfilament is inserted into the carotid artery and advanced to the middle cerebral artery, which reproduces the pathophysiology of ischemic stroke in humans (Sehara et al., 2018; Ye et al., 2025). Intraparenchymal injection of endothelin-1, a potent cerebrovascular constrictor, is another common choice for inducing focal ischemia (Yamazaki et al., 2021; Vinokurova et al., 2022; Zhou et al., 2025). In both models of ischemia, neurogenesis and oligodendrogenesis are promoted after reperfusion (Ceanga et al., 2021; Park et al., 2025).

Most studies relevant to cerebral ischemia are focused on neuronal death and neurogenesis; however. The post-ischemic function of oligodendrocytes is also important because they are responsible for axonal integrity. Not only neurons but also oligodendrocytes are vulnerable to ischemic injury owing to the high energy demand of axonal myelination (Hernández et al., 2021; Huang et al., 2023). In this review, we summarize the mechanisms and roles of both neurogenesis and oligodendrogenesis after ischemic injury, mainly on the basis of basic science studies.

## 2 Mechanisms and roles of post-ischemic neurogenesis

**Figure 1:**
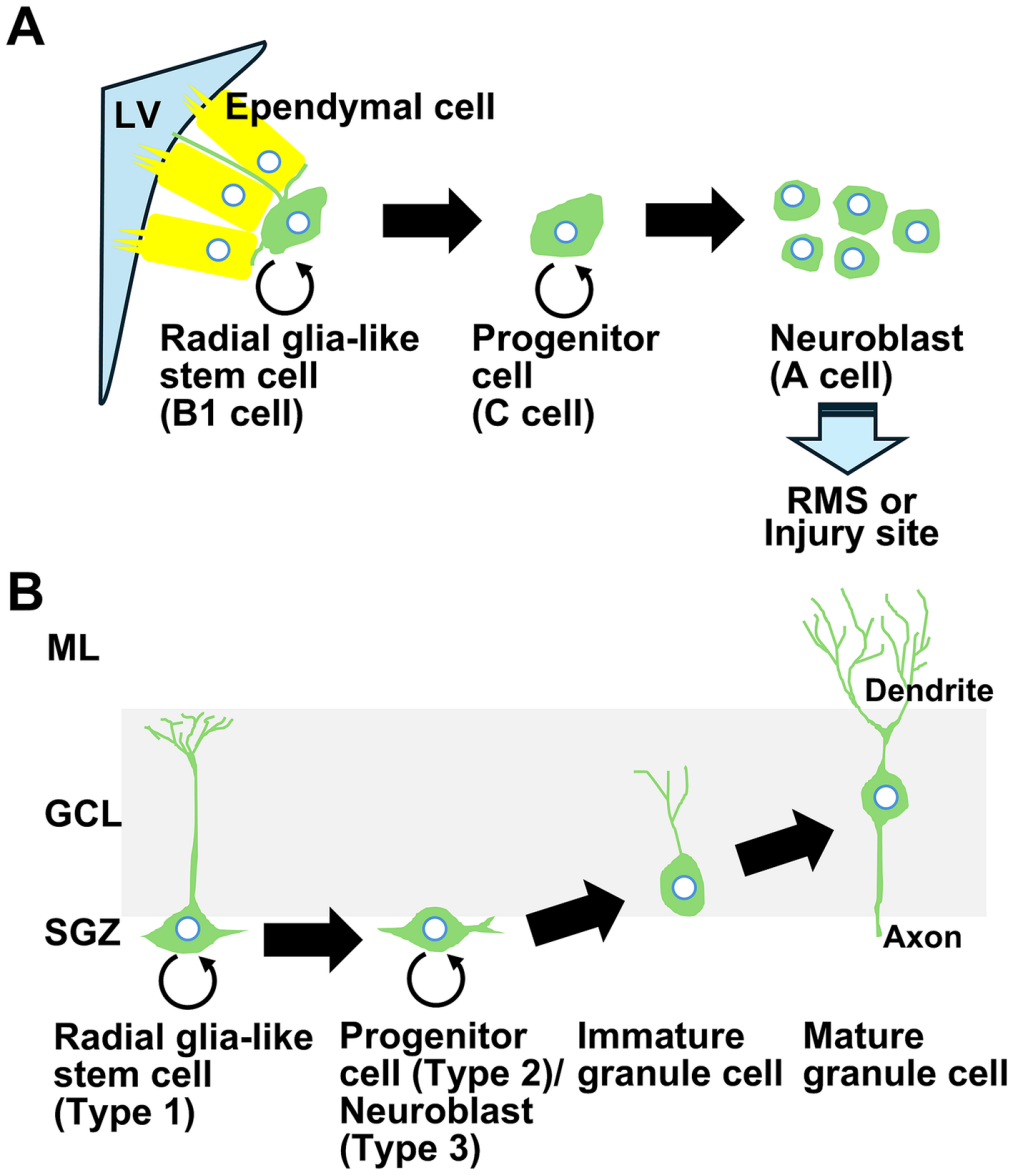
Neurogenesis in the subventricular zone of the lateral ventricle and the subgranular zone of the dentate gyrus. Neurogenesis persists throughout life both in the subventricular zone (SVZ) of the lateral ventricle (LV) and the subgranular zone (SGZ) of the dentate gyrus (DG). Both in focal and global models of ischemia, neurogenesis is abundantly upregulated in the SVZ and SGZ. In the SVZ, neural stem cells, called type B1 cells, give rise to neural progenitor cells, called type C cells, which generate a substantial supply of type A cells, which are characterized as neuroblasts and migrate to the olfactory bulb along the rostral migratory stream (RMS) under physiological conditions. After ischemia, neural stem cells in the SVZ are activated and migrate to the site of injury **(A)**. In the SGZ, radial glia-like cells, called type 1 cells, give rise to intermediate progenitors (type 2 cells) that further give rise to migratory neuroblasts (type 3 cells), which subsequently enter the maturation stage. This neurogenesis generates granule cells only in the granule cell layer (GCL), which receives input from the entorhinal cortex in the molecular layer (ML) of the DG. After ischemia, this neurogenesis promotes the recovery of cognitive function **(B)**.

It has been established that neurogenesis in the mammalian brain occurs in the subventricular zone (SVZ) of the lateral ventricle and the subgranular zone (SGZ) of the dentate gyrus throughout life (Altman and Das, 1965; Eriksson et al., 1998; Jurkowski et al., 2020; Geng et al., 2026). The fact that neurogenesis is upregulated in some brain insults such as ischemia, trauma, epilepsy, and Alzheimer’s disease is intriguing (Passarelli et al., 2022; Francis et al., 2025; Fu et al., 2025; Gage, 2025). After focal or global ischemia, neurogenesis is abundantly upregulated both in the SVZ and SGZ (Figure 1). Neurogenesis in the SVZ supplies new neurons to the injured region after focal ischemia, which promotes functional recovery (Jin et al., 2023). However, neurogenesis in the SGZ supplies new neurons solely to the granule cell layer of the dentate gyrus under both physiological and post-ischemic conditions (Kempermann et al., 2015; Passarelli et al., 2022). Neurogenesis in the dentate gyrus promotes the recovery of cognitive function after ischemic insult (Song et al., 2021; Sun et al., 2022) because the dentate gyrus forms one of the hippocampus subfields that plays an important role in learning and memory (Meier et al., 2020). It has been established that upregulation of endogenous neurogenesis promotes the recovery of brain function, although the precise mechanisms and roles remain unclear.

Growth factors and hormones have been extensively investigated in relation to post-ischemic neurogenesis. Growth factors, including brain-derived neurotrophic factor, ciliary neurotrophic factor, epidermal growth factor, erythropoietin, fibroblast growth factor-2, granulocyte-colony stimulating factor, insulin growth factor-1, and vascular endothelial growth factor, have been shown to enhance post-ischemic neurogenesis in rodent models (Matsuoka et al., 2003; Schäbitz et al., 2007; Sehara et al., 2007; Im et al., 2010; Popa-Wagner et al., 2010; Gonzalez et al., 2013; Kaneko et al., 2013; Shi et al., 2025). The hormones adiponectin, estradiol, ghrelin, growth hormone, melatonin, progranulin, testosterone, and vasoactive intestinal peptide have been reported to enhance neurogenesis in rodent models of focal ischemia (Suzuki et al., 2007; Chern et al., 2012; Fanaei et al., 2014; Yang et al., 2015; Sanchez-Bezanilla et al., 2020; Yu L. et al., 2023; Beuker et al., 2025).

Microglia play an instrumental role in the immune system of the brain. In SVZ, microglia reside alongside neural stem cells (NSCs) and are thought to regulate neurogenesis under homeostatic conditions (Zywitza et al., 2018). A recent report using single cell analysis revealed that ischemic insult leads to increased NSC proliferation and activation of microglial population in SVZ in a mouse model of focal ischemia (Nath et al., 2024). This result suggests that the altered microglia–NSC interactions limit the brain repair after ischemia.

Epigenetic modifications, such as DNA methylation, histone modification, and non-coding RNA, are likely important regulators of post-ischemic neurogenesis. Epigenetic modifications, which regulate neurogenesis during development, have been extensively investigated, although few studies have shown a direct relationship between epigenetic regulation and post-ischemic neurogenesis. Ten-eleven translocation methylcytosine dioxygenase 1 regulates gene expression through DNA demethylation, which was previously reported to enhance neurogenesis in the SGZ in a rat model of permanent bilateral common carotid artery occlusion-induced chronic hypoperfusion (Li et al., 2022). A recent report on single-cell nucleosome, methylome, and transcriptome analysis revealed that programming of striatal astrocytes into a neurogenic lineage after transient global ischemia requires methylome remodeling via DNMT3A (Kremer et al., 2024). Histone deacetylases (HDACs) regulate gene expression through chromatin remodeling by controlling the status of histone acetylation, and post-ischemic administration of a HDAC inhibitor enhanced neurogenesis in the SVZ and SGZ in a rat model of focal ischemia (Kim et al., 2009). In an examination of post-transcriptional regulation, systemic administration of miRNA-126-enriched exosomes enhanced neurogenesis in the periinfarct area in a mouse model of focal ischemia (Wang et al., 2020). Recently, we reported that virus-mediated knockdown of Ezh2, which suppresses gene expression by catalyzing trimethylation of lysine 27 of histone 3, abolished the post-ischemic increase in neurogenesis in the SGZ in a gerbil model of forebrain ischemia (Sehara et al., 2024).

Additional mechanisms by which post-ischemic neurogenesis improves the functional prognosis after ischemic insult remain to be revealed. An important question is how the newly generated neurons form novel neural circuits with the existing neurons.

## 3 Therapeutic perspective of ischemic stroke by oligodendrogenesis

**Figure 2:**
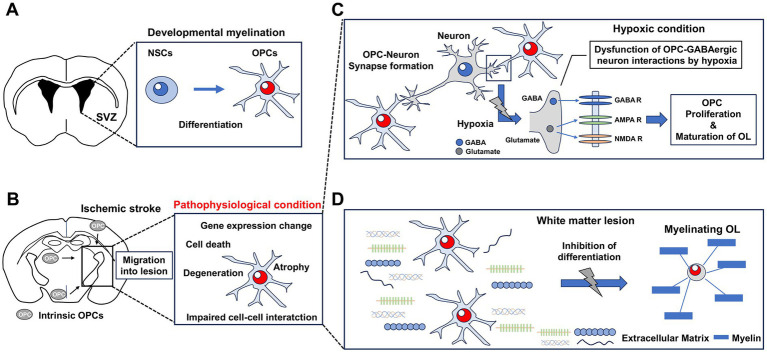
Oligodendrogenesis after ischemic stroke and inhibitors of white matter regeneration. **(A,B)** Oligodendrocyte progenitor cells (OPCs) are newly generated by oligodendrogenesis, which occurs during developmental myelination and after an ischemic stroke. **(A)** OPCs are derived from neural stem cells (NSCs) in the subventricular zone (SVZ) of the lateral ventricle, and then migrate to other areas of the brain. **(B)** OPCs derived from intrinsic cells that migrate from other brain regions and proliferate at the demyelinated lesion. The properties and morphology of OPCs change under pathophysiological conditions. **(C)** Gamma-aminobutyric acid (GABA) and glutamate are released to the GABA receptor, the *α*-amino-3-hydroxy-5-methyl-4-isoxazolepropionic acid (AMPA) receptor, and the N-methyl-D-aspartate (NMDA) receptor via OPC-neuron synapse formation. In hypoxic conditions, dysfunction of OPC-GABAergic neuron interactions is observed, as well as impaired OPC proliferation and OL maturation. **(D)** Some types of extracellular matrix in white matter lesions after ischemic stroke inhibit OPC differentiation and remyelination. These types of extracellular matrix include Chondroitin sulfate proteoglycans, hyaluronan, fibronectin, and type I collagen.

Ischemic stroke is one of the leading causes of white matter injury (Wang et al., 2016). Ischemia induces oligodendrocyte loss and demyelination by blood flow disturbances (Huang et al., 2023). White matter damage associated with cerebral ischemia causes cognitive impairment, motor paralysis, and depression (Wang et al., 2014; van Velzen et al., 2020; Filley, 2022; Carmichael and Llorente, 2023). Following a white matter injury, oligodendrocytes can spontaneously regenerate myelin around demyelinated axons (Nave and Werner, 2014; Bercury and Macklin, 2015; Yu Q. et al., 2023; Yamazaki and Ohno, 2024b). A recent report indicated that acute stroke patients with high white matter volume observed on synthetic MRI scans have a favorable prognosis (Toko et al., 2025). Therefore, myelin repair through oligodendrogenesis is important for functional recovery after an ischemic stroke. During remyelination, newly generated oligodendrocyte progenitor cells (OPCs) differentiate into mature oligodendrocytes in the white matter lesions (Baaklini et al., 2019; Franklin and Simons, 2022; Yamazaki et al., 2023; Yamazaki and Ohno, 2025a). These OPCs include those derived from NSCs in the SVZ of the lateral ventricle, as well as those derived from intrinsic cells that migrate from other brain regions, and proliferate at the demyelinated lesions (Maki et al., 2013; Zhang et al., 2013). During the early stages of postnatal development, the SVZ is an important area for OPC production in the brain (Ivanova et al., 2003; Suzuki and Goldman, 2003; Kaneko et al., 2013). Activated microglia secrete cytokines in the SVZ, promoting neurogenesis and oligodendrogenesis in the early postnatal period in rodents (Shigemoto-Mogami et al., 2014). However, chronic or excessive neuroinflammation via microglia and astrocyte activation and infiltration of peripheral T and B cells impaired oligodendrogenesis and myelination (Planas, 2024; Festa et al., 2025). In hypoxic conditions during the perinatal stage, activated microglia release inflammatory cytokines and follistatin, an activin-A inhibitor that regulates remyelination (Dillenburg et al., 2018; Zheng et al., 2021). Dysregulation of the Wnt/*β*-catenin signaling pathway in microglia is also involved in white matter pathology (Van Steenwinckel et al., 2019). Reactive astrocytes secrete the inflammatory molecules tumor necrosis factor-*α*, interleukin-1α, and C1q, as well as prostaglandin E2 (PGE2), through the upregulation of cyclooxygenase-2 (COX2) (Shiow et al., 2017; Renz et al., 2024). The COX2-PGE2 signaling inhibits white matter regeneration (Renz et al., 2024). Previous reports have clarified that OPCs are produced in the SVZ throughout adulthood and migrate to produce myelin in various brain regions (Levison and Goldman, 1993; Nait-Oumesmar et al., 1999; Picard-Riera et al., 2002; Menn et al., 2006; Kaneko et al., 2013) (Figure 2A). Additionally, ischemic stroke induces the migration of intrinsic OPCs into the lesion (Maki et al., 2013; Zhang et al., 2013) (Figure 2B). However, despite the accumulation of OPCs in white matter lesions of cerebral ischemia, OPC differentiation and remyelination are inhibited (Wang et al., 2016; Yamazaki et al., 2021; Huang et al., 2023; Yamazaki and Ohno, 2024a). Therefore, induction of oligodendrogenesis alone is insufficient for white matter regeneration, and remyelination is necessary to induce functional recovery after an ischemic stroke. Additionally, the specific properties of OPCs have recently been clarified. OPCs not only contribute to remyelination, but they are also involved in neural circuit development and axon remodeling (Buchanan et al., 2023; Yi et al., 2023). OPCs have a role in synapse formation and synapse engulfment, and play an important function in the remodeling of neural circuits (Bergles et al., 2000; Mount et al., 2019; Auguste et al., 2022; Buchanan et al., 2022). Furthermore, OPCs have been shown to promote white matter angiogenesis, and it has been reported that OPCs present antigens to cytotoxic CD8^+^ T cells in inflammatory demyelination, which results in OPC death (Yuen et al., 2014; Falcão et al., 2018; Kirby et al., 2019). The pathophysiology of OPCs is characterized by cell degeneration and death, atrophy with loss of function, gene expression changes, and impaired interactions with neurons and glial cells (Buchanan et al., 2023; Yi et al., 2023) (Figure 2B). OPCs express receptors for glutamate and GABA, and interact with neurons by forming synapses (Balia et al., 2017; Moura et al., 2021). In hypoxia, synaptic input to OPCs via GABA_A_ receptors is impaired (Zonouzi et al., 2015), suggesting that dysfunction of OPC-GABAergic neuron interactions impairs regeneration (Figure 2C). Therefore, OPCs may play an important role in neural circuit reorganization after ischemic stroke. Overall, OPCs may also play a pivotal role in regeneration processes beyond remyelination.

Previously, it has been suggested that promoting white matter regeneration may induce functional recovery in rodent stroke models (Youssef et al., 2021). However, previous research using rodents has not yet led to the development of effective treatments. Removal of factors that inhibit remyelination may represent a new treatment strategy for inducing remyelination by oligodendrocytes. For example, it was recently reported that the extracellular matrix deposited in white matter lesions inhibits remyelination (Figure 2D) (Sobel and Ahmed, 2001; Back et al., 2005; Ghorbani and Yong, 2021; Yamazaki et al., 2025). These findings have drawn attention to extracellular matrices that accumulate in white matter lesions as a potential new therapeutic target for oligodendrogenesis and remyelination. These potential targets include chondroitin sulfate proteoglycans (CSPGs), hyaluronan, fibronectin, and fibrinogen (Back et al., 2005; Lau et al., 2012; Stoffels et al., 2013; Petersen et al., 2017). Therapeutic studies targeting the receptors and downstream signals of these molecules have been conducted using rodent models of white matter injury (Shen et al., 2009; Davalos et al., 2012; Lang et al., 2015; Keough et al., 2016; Feliú et al., 2017; Petersen et al., 2017; Qin et al., 2017; Luo et al., 2018). Additionally, it has recently been reported that type I collagen, a representative extracellular matrix, is deposited in the white matter lesions of stroke patients. Furthermore, this study clarified that the differentiation of oligodendrocytes is inhibited in the presence of type I collagen in white matter lesions (Yamazaki et al., 2025; Yamazaki and Ohno, 2025b). It may be possible to induce oligodendrocyte differentiation and achieve remyelination not only by promoting oligodendrogenesis, but also by removing factors that inhibit white matter regeneration.

However, the limitations using rodent models of white matter injury need to be discussed. The proportion of white matter in the human brain is significantly higher than in rodents, accounting for nearly 50% of the total brain volume (Semple et al., 2013; Ventura-Antunes et al., 2013; Guan and Kong, 2015). Differences in white matter volume may alter the ratio and properties of oligodendrocytes present in the brain and the CNS environment. A study using white matter injury models showed that long-term administration of clemastine, a drug that promotes remyelination is necessary to induce white matter regeneration in rabbits compared to mice (Cooper et al., 2024). Furthermore, aging impairs remyelination after white matter damage (Cantuti-Castelvetri et al., 2018; Neumann et al., 2019). Recently, it was reported that oligodendrocytes that were newly generated in aged mice exhibit impaired differentiation and morphological abnormalities (Looprasertkul et al., 2025).

Interestingly, single-cell RNA sequencing analysis has identified various subtypes of human oligodendrocytes in neurodegenerative diseases, and these oligodendrocytes have complex gene expression profiles (Jäkel et al., 2019; Osanai et al., 2022; Seeker and Williams, 2022; Weng et al., 2025). Therefore, species differences and oligodendroglial heterogeneity may hinder the translation of basic research results into clinical applications.

## 4 Conclusion

In this review, we summarized the roles and mechanisms of neurogenesis and oligodendrogenesis that have been reported to date. Previous reports using rodent models have identified many factors that promote or inhibit functional recovery after cerebral ischemia and have discussed the importance of neurogenesis and oligodendrogenesis. However, there is currently no available therapy targeting neurogenesis and oligodendrogenesis after stroke. Therefore, to promote functional recovery following stroke, a precise understanding of the molecular mechanisms of neurogenesis and oligodendrogenesis is necessary. To our knowledge, a direct regulatory relationship between neurogenesis and oligodendrogenesis after ischemia has not yet been reported; however, recent advancements in methylome and transcriptome techniques may shed light on this relationship. Future studies are required to gain a deeper understanding of the communication between neurons and glial cells and the lesion environment to induce neural circuit reorganization and remyelination following neurogenesis and oligodendrogenesis. In particular, the function and interaction of recently discovered disease-associated oligodendrocytes with neurons and glia remain unclear. Therefore, these oligodendrocytes may expand our understanding of ischemia. These studies may lead to the identification of factors that inhibit neural circuit reorganization and remyelination, potentially paving the way for novel therapeutic strategies.
