# Correction: Post-ischemic modification of neurogenesis and oligodendrogenesis in rodent models

**DOI:** 10.3389/fncir.2026.1863549

**Published:** 2026-05-05

**Authors:** Yoshihide Sehara, Shinya Mochizuki, Reiji Yamazaki

**Affiliations:** 1 Division of Genetic Therapeutics, Center for Molecular Medicine, Jichi Medical University, Shimotsuke, Japan; 2 Department of Cell Biology and Anatomy, Jichi Medical University, Shimotsuke, Japan; 3 Department of Anatomy, Division of Histology and Cell Biology, School of Medicine, Jichi Medical University, Shimotsuke, Japan

**Keywords:** ischemia, neural stem/progenitor cells, neurogenesis, oligodendrogenesis, stroke

An incorrect number was provided for #JP24wm0624321. The correct number is JP24wm0625321.

The original version of this article has been updated.

